# Adverse drug reactions reporting practice and associated factors among community health extension workers in public health facilities, Southwest, Nigeria

**DOI:** 10.11604/pamj.2021.40.165.28574

**Published:** 2021-11-17

**Authors:** Waheed Adeola Adedeji, AbdulKabir Babajide Adegoke, Fatai Adewale Fehintola

**Affiliations:** 1 Department of Pharmacology and Therapeutics, University of Ibadan, Ibadan, Oyo State, Nigeria,; 2 Department of Clinical Pharmacology, University College Hospital, Ibadan, Oyo State, Nigeria,; 3 Department of Community Health and Primary Care, Lagos University Teaching Hospital, Idi-Araba, Lagos, Nigeria

**Keywords:** Attitude, community health workers, adverse drug reactions, health facilities, knowledge, Nigeria

## Abstract

**Introduction:**

timely adverse drug reactions (ADRs) reporting has contributed immensely towards public health safety. Community health extension workers (CHEWs) provides basic medical care in rural areas. This study assessed the knowledge, attitude, practice, and determinants of ADRs reporting among CHEWs in public health institutions, Southwest, Nigeria.

**Methods:**

a cross-sectional survey of 333 CHEWs randomly selected from public health facilities using self-administered questionnaires. The questionnaire sought information on the knowledge, attitude and practice of CHEWs towards ADRs reporting. The knowledge and attitude questions were scored and categorized. The association between dependent and independent variables assessed with bivariate and multivariate logistic regressions, and p < 0.05 considered statistically significant.

**Results:**

among 333 respondents, 205 (61.6%) had encountered patients with ADRs but only 26 (12.6%) had reported it with yellow forms. About half, 169 (50.8%), and 191 (57.4%) respondents had a positive attitude and inadequate knowledge of ADRs reporting respectively. Sex (aOR: 2.84, 95% CI: 2.10-7.10; p < 0.0001), working in Ogbomoso area (aOR: 3.3, 95% CI: 1.34-8.21; p=0.01), and training (aOR: 2.01, 95% CI: 1.20-3.42; p = 0.01) were factors associated with adequate knowledge. The determinant of ADRs reporting was training (aOR: 3.63, 95% CI: 1.13-11.63; p = 0.03).

**Conclusion:**

though CHEWs had a slightly positive attitude, they had inadequate knowledge and poor ADRs reporting. The determinant of inadequate ADRs reporting knowledge and under reporting was lack of training. There is an urgent need for educational intervention programmes towards improving knowledge and practices of ADRs reporting among CHEWs.

## Introduction

Adverse Drug Reactions (ADRs) constitute an important cause of morbidity and mortality worldwide [1] and have been reported as the sixth leading cause of death in the U.S after heart disease, cancer, stroke, pulmonary disease, and accidents [2]. In addition to potentially causing ill-health, ADRs impose a heavy economic loss on nations [3,4]. Despite the burden of ADRs, many times it is either not recognised as the cause of the patient´s problem, or when recognised, it may not be reported by health professionals [5-7]. The most common method of reporting ADRs worldwide is spontaneous reporting, which is done through pharmacovigilance [8-11]. However, the major problem of spontaneous ADR reporting worldwide is under reporting [5], but it is probably worse in developing countries. Inappropriate use of drugs is common in Africa [12]. It is expected that the ADRs emanating from the continent would be high. Contrarily, ADR reports from Africa represent the least of the report to the VigiBase [13,14]. The decision on post-marketing withdrawal of medicines relies on the ADRs reported, and consequently, the continent continually has the least post-marketing withdrawal of unsafe medicines [15].

The Pharmacovigilance activities in Nigeria are coordinated by the National Pharmacovigilance Centre (NPC), at the National Agency for Food and Drug Administration and Control. All healthcare providers are to report any observed ADRS as part of their professional responsibility to NPC. NPC receive, collate and analyze submitted ADRs and transmit such to the WHO Uppsala Monitoring Centre [16,17]. Studies have shown that between 2.0 and 7.3% of Physicians practising in urban areas of Nigeria reported ADRs [18-21]. Similarly, low reporting rates have been reported among health care workers in urban areas in Nigeria. Also, there were inadequate knowledge and a negative to a moderately positive attitude of ADR reporting among health care professionals [22-27]. In Nigeria, the majority of health care professionals work in urban centres. The rural areas are devoid of health care workers and facilities. Most of the health facilities in the rural areas are manned by the Community Health Extension Workers (CHEWs). CHEWs provide support in the management of minor medical illnesses, antenatal care, routine and supplementary immunization services and referrals. NPC encourages all health workers to report suspected ADRs [17], but little is known about ADRs reporting among CHEWs. This study assessed the attitude, knowledge and practice, and determinants of ADR reporting among CHEWs in public health institutions in Oyo State, Southwest, Nigeria.

## Methods

**Study design:** a cross-sectional study was conducted among CHEWs working in public health institutions, in Oyo State, Southwest Nigeria, between April and September 2014.

**Setting:** the capital of Oyo State is Ibadan. There are 33 Local Governments (LGs) in the state. The population of Oyo State according to the 2006 National census was 5,380, 894. The state was divided into five geopolitical zones: Zone I (Ibadan Area)-comprising 11 LGs, Zone II (Okeogun Area)-10 LGs, Zone III (Ogbomoso Area)-5 LGs, Zone IV (Oyo Area)-4 LGs and Zone V (Ibarapa Area)-3 LGs. There were 678 public health institutions in the state, comprising 517 primary health centres, 45 secondary health facilities, and 6 tertiary health centres.

**Participants:** they were CHEWs randomly selected from public primary and secondary health facilities in Oyo State. The PHC coordinators (for LGs) and hospital heads (secondary /general hospitals) were requested to randomly select one CHEW per health facility, as part of health care workers for the Malaria Action Program for States (MAPS) training. MAPS was case management training for acute uncomplicated malaria in Oyo State sponsored by FHI 360 and took place in all five zones. All CHEWs who provided verbal informed consent were asked to complete a self-administered study questionnaire on the first day, and submit the questionnaire the same day before leaving the venue of the training. A trained research assistant was employed for the research. And together with the principal investigator (PI) were available to clarify any question during the completion of the questionnaire.

**Data sources/measurement:** the study self-administered questionnaire included four sections and was adapted from previous studies [20,21,28]. Section A contains information on the socio-demographic characteristics of the participants, section B, knowledge of ADR reporting, section C, attitude of ADR reporting and section D, practices of ADR reporting and an open question on suggested ways of improving ADR reporting. Twenty questions were used to assess the knowledge of ADRs reporting. The knowledge questions were Yes/No with one mark allocated for one correct response giving a total score of 20 marks. The attitude questions comprising of 15 questions and using a 3-point Likert scale (agree, neutral and disagree) was used to measure the participants´ level of agreement with the survey questions. The reliability test (Cronbach alpha) on SPSS version 21 for the knowledge questions/scale and attitude questions/scale was 0.88 (0.86, 0.90) and 0.87 (0.84, 0.89) respectively. The normality plot test (Kolmogorov-Smirnov and Q-Q plot) of both the knowledge and attitude score were not normally distributed. For the knowledge score, a score of more than or equal to 12 was ranked as adequate knowledge while a score of less than 12 was ranked as inadequate knowledge. For the attitude, a score greater than or equal to 32 (median score) was considered a positive attitude while a score of less than 32 was ranked as a negative attitude.

**Variables:** dependent variables- overall knowledge and attitude of CHEWs about ADR reporting, and ADRs reporting with form. Independent variables- Age, sex, years of professional experience, marital status, attended training on ADRs reporting, level of practice, and geographical zones.

**Study size:** the sample size was calculated using the Leslie Kish formula, n = pqZ^2^/d^2^ [29]. Assuming 50% of the respondents will have adequate knowledge of ADRs reporting, the critical value for α at p < 0.05 of 1.96, precision (d)=5%. After adjustments for the population (number of CHEWs in the state=1,121) and 10% non-response, a minimum sample size of 317 was obtained.

**Statistical methods:** data was analysed using IBM-SPSS version 22. The continuous variables like age, years of professional experience, knowledge, and attitude score were summarized with mean (± standard deviation), or median (range) if not normally distributed. The categorical variables like sex, marital status, level of practice, geographical zones, ever received training, reporting ADRs with ADR form, knowledge, and attitude (categorized) were summarised using frequency and proportion. Association between knowledge (adequate and inadequate knowledge), attitude (positive and negative), reporting ADRs with forms and selected independent variables were assessed with odds ratios and Chi-square. Statistically significant variables in the bivariate analyses were included in multivariate analyses. Multivariate analyses were performed with binary logistic regression. The level of statistical significance was set at < 0.05.

**Ethical consideration:** the study was approved by the University of Ibadan/University College Hospital ethical review committee (UI/EC/12/0418). Verbal informed consent was obtained from the participants.

## Results

**Participants:** a total of 400-questionnaires were distributed to the CHEWs, of which 333 were completed and returned within the stipulated time, given a response rate of 83.3%. The majority of survey respondents were female (78.4%) and the mean age of the respondents was 43.5 (±8.3) years. The median (range) years of professional experience was 18 (1 to 40) years. One-third of the respondents have ever received training on ADRs reporting (Table 1).

**Table 1:** sociodemographic characteristics of community health extension workers in public health facilities, South-West Nigeria

Variables	Frequency (%)
**Sex**	
Male	72(21.6)
Female	261(78.4)
**Age group**	
20-29	15(4.5)
30-39	95(28.5)
40-49	110(33.0)
50-69	113(33.9)
**Marital Status**	
Single	18(5.4)
Married	301(90.4)
Formerly married*	14(4.2)
**Year of Practice**	
1-10	79(23.9)
11-20	121(36.5)
21-30	90(27.2)
31-40	41(12.4)
**Level of practice**	
Primary	183(55.0)
Secondary	109(45.0)
**Geopolitical zones**	
Ibadan	109(32.7)
Ogbomoso	42(12.6)
Ibarapa	68(20.5)
Okeogun	39(11.7)
Oyo	75(22.5)
**Ever been trained on reporting ADRs**	
Yes	102(30.6)
No	231(69.4)

* Formerly married include widowed, divorced and separated

**Knowledge of community health extension workers on ADRs reporting:** the majority of the respondents 246 (73.9%) knew that ADRs constitute an important problem in the medical practice. Awareness of the existence of the National Pharmacovigilance Centre (NPC) in Nigeria was low, 156 (46.8%) and only 63 (18.9%) respondents knew the location is in Abuja. The knowledge of ADRs to the agents (drugs, vaccines and medical devices etc.) to be reported was generally inadequate among the respondents. The median (range) proportion of the respondents who knew the ADRs to the agents to be reported was 41 (31.5 to 49.2). More than 70% of the respondents knew that suspected ADRs, confirmed ADRs, serious reactions and the reactions to the newly introduced drugs in the market should be reported. The mean knowledge score of the respondents was 10.2 (±5.3) while the median (range) score was 10 (0 to 20). One hundred and forty-two (42.6%) and 191 (57.4%) respondents had adequate and inadequate knowledge of ADRs reporting respectively.

**Factors associated with community health extension workers knowledge of ADRs reporting:** males had more knowledge of ADRs reporting than females, COR: 4.5, 95% CI 2.30-8.01; p<0.0001). Respondents who have ever had training on ADRs reporting were 1.83 times more likely to have adequate knowledge of ADRs reporting than those who have not had training. Those who are in secondary health facilities were about 47 times more likely to have adequate knowledge than those in the primary health care facilities. The determinant of adequate knowledge of ADRs reporting were male gender, respondents from Ogbomoso zone and ever received training on ADRs reporting (Table 2).

**Table 2:** factors associated with the knowledge of community health extension workers on adverse drug reactions reporting, South-West, Nigeria

Variables	Adequate Knowledge Number (%)	Inadequate Knowledge Number (%)	COR (95% CI)	p-value	aOR (95% CI)	P-value
**Sex**						
Male	51(70.8)	21(29.2)			2.84(2.10, 7.10)	<0.0001
Female*	91(3.9)	170(65.1)	4.5(2.30, 8.01)	<0.0001	1	
**Level of practice**						
Primary	63(34.4)	120(65.6)			0.58(0.34, 1.00)	0.05
Secondary*	79(52.7)	71(47.3)	0.47(0.30, 0.73)	0.001	1	
**Geopolitical zones**						
Ibadan*	48(44.0)	61(56.0)			1	
Ogbomoso	8(19.0)	34(81.0)		0.007	3.3(1.34, 8.21)	0.01
Ibarapa	36(52.9)	32(47.1)			1.19(0.56,2.51)	0.656
Okeogun	14(35.9)	25(64.1)			0.99(0.44, 2.3)	0.977
Oyo	36(48.0)	39(52.0)			1.05(0.55, 2.01)	0.877
**Training on ADRs**						
Yes	54(52.9)	48(47.1)			2.01(1.18, 3.42)	0.010
No*	88(38.1)	143(61.9)	1.83(1.14, 2.93)	0.016	1	
**Marital Status**						
Single	8(44.4)	10(55.6)				
Married	134(42.5)	181(57.5)	1.08(0.42, 2.80)	0.53		
**Age**						
<40 years	49(44.5)	61(55.5)				
≥ 40 years	93(41.7)	130(58.3)	1.12(0.71, 1.78)	0.639		
**Years of practice**						
≤ 10 years >10 years	39(48.1) 103(40.9)	42(51.9) 149(59.1	1.34(0.81, 2.2)	0.302		
						

* Reference categories, COR: crude odds ratio, aOR: adjusted odds ratio, 95% CI: 95% confidence interval

**The attitude of community health extension workers on ADRs reporting:** the majority had unfavourable attitudes to most of the questions tested except to those on the usefulness of ADRs reporting information, 205 (61.6%), reporting preventing respondents from publishing a case series of ADRs, 175 (52.6%) and professional obligation of ADRs reporting, 221 (66.4%). The mean attitude score was 32.1 (±7.4) while the median (range) was 32 (15 to 45). About half 169 (50.8) had a positive attitude to ADRs reporting.

**Factors associated with community health extension Workers´ positive attitude on ADRs reporting:** the factors that were significantly associated with positive attitudes towards ADRs reporting were male gender and working in Okeogun zone. The determinant of positive attitudes towards ADRs reporting was respondents from the Okeogun zone, aOR: 4.51, 95% CI 1.9-11.01; p=0.001 (Table 3).

**Table 3:** factors associated with positive attitude of community health extension workers on adverse drug reactions reporting, South-west Nigeria

Variables	Positive Attitude Number (%)	Negative Attitude Number (%)	COR (95% CI)	P-value	aOR (95% CI)	P-value
**Sex**						
Male	45(62.5)	27(37.5)	1.8(1.1, 3.2)		1.57(0.87, 2.83)	0.134
Female*	124(47.5)	137(52.5)		0.03	1	
**Geopolitical** zones						
Ibadan*	62(56.9)	47(43.1)			1	
Ogbomoso	18(42.9)	24(57.1)		<0.001	1.70(0.8, 3.5)	0.178
Ibarapa	32(47.1)	36(52.9)			1.84(0.91, 3.8)	0.165
Okeogun	8(20.5)	31(79.5)			4.51(1.9, 11.01)	0.001
Oyo	49(65.3)	26(34.7)			0.73(0.39, 1.4)	0.336
**Marital Status**						
Single	9(50)	9(50)				
Married	160(50.8)	155(49.2)	0.97(0.40, 2.51)	0.569		
**Age**						
<40 years	61(55.5)	49(44.5)		0.245		
≥ 40 years	108(48.4)	115(51.6)	1.33(0.84, 2.1)			
**Level of practice**						
Primary	86(47.0)	97(53.0)	0.72(0.464, 1.104)	0.08		
Secondary	83(55.3)	67(44.7)				
**Years of practice**						
≤ 10 years	43(53.1)	38(46.9)	1.1(0.69, 1.90)	0.702		
>10 years	126(50.0)	126(50.0)				
**Training on ADRs**						
Yes	60(58.8)	42(41.2)	1.6(0.99, 2.60)	0.057		
No	109(47.2)	122(52.8)				

* Reference categories, COR: crude odds ratio, aOR: adjusted odds ratio 95% CI: 95% confidence interval

**Practices of community health extension workers regarding ADRs reporting:** about two-third, 205 (61.6%) respondents have observed patients with ADRs, but only 26 (12.6%) of the respondents had reported with ADRs forms. When asked about which drugs were suspected or confirmed as the cause of the observed ADRs, 23 respondents identified chloroquine, 15 identified co-trimoxazole, 6 identified procaine penicillin, 5 identified ivermectin, and 2 identified multiple medications. One hundred and ninety-two respondents (57.7%) indicated that they always consider the possibility of ADRs before prescribing, dispensing or administration of drugs. Only about one-third of the respondents have ever received training on ADRs reporting (Table 4).

**Table 4:** adverse drug reactions reporting practices of the community health extension workers in public health facilities, South-west Nigeria

Questions	Category	Frequency	Percentage
Have you ever encountered patients with ADRs in your practice?	Yes	205	61.6
	No	128	38.4
If yes, have you ever reported it with ADRs form?	Yes	26	12.6
	No	179	87.4
When last did you encounter the patients with ADRs?	Last one month	8	3.9
	Last one year	170	82.9
	Last two years	27	13.2
What are drugs suspected to cause the ADRs encountered? N=57	Chloroquine	23	40.4
	Amodiaquine	6	10.5
	Cotrimoxazole	7	12.3
	Procaine penicillin	6	10.5
	Antibiotics*	8	14.0
	Ivermectin (MectizanR)	5	8.8
	Drugs’interaction	2	3.5
How do you manage the ADRs encountered? N=53	Discontinuation of suspected drug	9	17.0
	Discontinuation of suspected drug and administration of hydrocortisone	11	20.8
	Hydrocortisone administration	4	7.5
	Hydrocortisone and intravenous fluid	8	15.1
	Chlorpheniramine administration	12	22.6
	Intravenous fluid	9	17.0
Do you consider the possibility of ADRs before prescribing, dispensing, or administering drugs?	Always	192	57.7
	Sometimes	91	27.3
	Never	50	15.0
Do you obtain information on ADRs regularly?	Yes	132	30.6
	No	231	69.4
If yes, sources of regular information on ADRs N=132	Ministry of health drug	43	32.8
	Information bulletin	55	42.4
	Drug information sheet	17	12.4
	Textbooks on drugs and therapeutics	13	9.5
	Drug sales representatives scientific journals	4	2.9
Have you ever been trained on ADRs reporting	Yes	102	30.6
	No	231	69.4
If yes, sources of training N=59	School	10	17.0
	Part of routine immunization training	8	13.5
	National malaria control program	33	56.0
	Seminar/workshop	7	11.8
	National youth service corps (NYSC)	1	1.7

**Factors influencing ADRs reporting by community health extension workers:** males were 2.73 times more likely to report ADRs than females. Other factors that were significantly associated with reporting ADRs were age less than 40 years, less than or equal to 10 years of professional experience, and those who have had training on ADRs reporting. The only determinant of ADRs reporting was training, aOR: 3.63, 95% CI 1.13-11.63; p=0.01 (Table 5).

**Table 5:** factors associated with adverse drug reactions reporting by the community health extension workers, South-west Nigeria

Variables	Reporting ADR Number (%)	Not Reporting ADR Number (%)	COR (95% CI)	p-value	AOR (95% CI)	P-value
**Sex**						
Male	9(34.6)	17(65.4)			2.79(0.65, 11.93)	
Female	13(16.3)	67(83.8)	2.73(1.001, 7.44)	0.045	1	0.166
**Age**						
<40 years	14(35.9)	25(64.1)	4.13(1.54,	0.004	6.38(0.78, 51.95)	0.083
≥ 40 years	8(11.9)	59(88.1)	11.10)		1	
**Years of practice**						
≤ 10 years	9(34.6)	17(65.4)	2.73(1.001,	0.046	1.57(0.31, 7.95)	
>10 years	13(16.3)	67(83.8)	7.44)		1	0.588
**Training on ADRs**						
Yes	13(37.1)	22(62.9)	4.07(1.53,	0.005	3.63(1.13, 11.63)	0.030
No	9(12.7)	62(87.3)	10.84)		1	
**Marital Status**						
Single	1(11.1)	8(88.9)	0.45(0.05, 3.82)	0.404		
Married	21(21.6)	76(78.4)				
**Level of practice**						
Primary	7(18.9)	30(81.1)	0.84(0.31, 2.29)	0.470		
Secondary	15(21.7)	54(78.3)				
**Geopolitical zones**						
Ibadan	6(18.8)	26(81.3)				
Ogbomoso	4(30.8)	9(69.2)		0.452		
Ibarapa	7(25.9)	20(74.1)				
Okeogun	0(0.0)	9(100.0)				
Oyo	5(20.0)	20(80.0)				
**Knowledge of ADRs reporting**						
Adequate	13(37.1)	48(78.7)	1.08(0.42, 2.81)	0.534		
Inadequate	9(20)	36(80.0)				
**Attitude to ADRs reporting**						
Positive	14(21.5)	51(78.5)	1.13(0.43, 3.0)			
Negative	8(19.5)	33(80.5)				

* Reference categories, COR: Crude Odds Ratio, aOR: Adjusted Odds Ratio 95% CI:95% Confidence Interval

## Discussion

The main method of preventing ADRs worldwide is through spontaneous reporting [10,11,30], the limiting factor of which is underreporting. Under-reporting of ADRs is related to knowledge, attitude and practice of health workers towards ADRs reporting [20,28,31,32]. This study assessed the knowledge, attitude, practice and determinants of ADRs reporting by CHEWs in public health facilities. The respondents had a slightly positive attitude, inadequate knowledge and poor ADRs reporting. The determinant of ADRs reporting among the respondents was training. This study revealed inadequate knowledge of ADRs reporting by the respondents. Only one-third of the respondents have ever received training on ADRs reporting, and this may be an important contributory factor. Pharmacovigilance is not included in the curricula of many training schools in developing countries, and healthcare professionals only become aware during practice. Moreover, the majority of the respondents work in primary health care facilities where exposure to information on ADRs reporting are lacking. Studies among health care workers in Nigeria [20,21,24,28] and other developing countries[32-34] have reported inadequate knowledge of ADRs reporting by health care workers. However, a study in a tertiary centre in Ibadan, Nigeria, reported adequate knowledge of ADRs reporting by physicians [35]. The centre has a functioning Pharmacovigilance Committee, which coordinates pharmacovigilance training for health care workers and hospital ADRs reporting. Though this may not account entirely, there is a difference in knowledge gap among different health professionals.

The determinants of adequate knowledge included male gender, respondents working in Ogbomoso zone and training on ADRs reporting. The reason for more respondents with adequate knowledge among males than females could not be immediately proffered. Males CHEWs are few and competition in a female-dominated profession may be an important reason. Contrarily, studies have reported female health workers as having adequate knowledge of ADRs and reporting than males [36-38]. Respondents who have received training on ADRs reporting were more likely to report an observed ADR than those who have not received any training. Studies in African countries have reported education and training as a means of improving knowledge and reporting of ADRs [20,31,32]. Also, studies have reported the impact of educational interventions on the improvement in the knowledge, attitude and practices of health professionals on ADRs reporting [27,39,40]. The reason for the findings of adequate knowledge of ADRs reporting among respondents from Ogbomoso zone could not immediately be ascertained, however, it may relate to the positive attitude of the heads of health facilities towards ADRs reporting.

The slightly positive attitudes of respondents towards ADRs reporting does not translate to increase ADRs reporting. Studies have reported a positive attitude with poor ADRs reporting [32,33,36]. A review study had reported attitudes towards ADRs reporting as a determinant of under-reporting of ADRs worldwide [5]. Males were more likely to have a positive attitude than females, but the statistical significance was not sustained after multivariate analysis. The only determinant of a positive attitude toward ADRs reporting was working in Okeogun zone. This may be related to the attitude of the leadership of the various health facilities in the zone. However, this was not assessed in this study. Although majority have observed ADRs, only a few had reported it with the ADRs form. A major factor that may be implicated is the inadequate knowledge of ADRs reporting. Likewise, studies have reported low ADRs reporting in Nigeria [19-21, 23] and other developing countries [31-34, 38, 41] among health care professionals. Moreover, despite the National guidelines for the management of malaria, majority of the ADRs reported by the respondents were to antimalarial monotherapy-chloroquine or amodiaquine. This highlights the disparity between the guideline and practice especially at the primary health care level. In Nigeria, presumptive treatment of malaria for any case of fever is common, especially at the primary health care level. Our findings are in agreement with that of Sevene et al. in rural districts of Mozambique where the most implicated drugs were antimalarial and cotrimoxazole [42]. Cotrimoxazole is commonly prescribed at the PHC level and a commonly self-medicated antibacterial agent due to its affordability and availability. It has been implicated as the most common cause of life-threatening/serious ADRs like Steven-Johnson´s syndrome (SJS) and toxic epidermal necrolysis (TEN) in sub-Saharan Africa [43].

The only determinant of ADRs reporting was the lack of training on ADRs reporting. Similarly, studies have reported a lack of training as a major deterrent to ADRs reporting [20,31,32,44]. Contrarily, studies have reported female gender [36-38] higher level of education [38], higher working experience [32,35,38,44] and the existence of ADRs reporting forms [23,32,38], as positively influencing ADRs reporting. However, our study found male respondents to be more likely to report ADRs than females. Males may have more aptitude to report ADRs than their females´ counterparts as against what was mostly reported by previous studies. Also, respondents aged less than 40 years and those with less than or equal to 10 years of professional experience were more likely to report ADRs. However, these factors were not sustained after multivariate analysis. The fact that studies used different cut-offs for years of professional working experiences may explain the dissimilar findings. Contrary to a study in Ethiopia [38], our study did not obtain the level of education of the respondents, rather they were treated as one professional cadre. The limitations of this study included the possibility of selection bias, reporting and recall biases among the respondents. There was a long delay in publishing these results. To the best of our knowledge, this is the first study on knowledge, attitude and practices of ADRs reporting among CHEWs. The importance cannot be overemphasized in developing countries considering the shortage of health care workers and the continuing need for new vaccines and hence adverse events following immunization (AEFI) and drugs (hence ADRs) in the midst of emerging and re-emerging infectious diseases, including COVID-19. This study provides baseline information for designing an educational intervention for improving ADRs and AEFIs reporting by CHEWs.

## Conclusion

Community health extension workers (CHEWs) working in the public health facilities of Oyo State, Southwestern Nigeria had inadequate knowledge and poor ADRs reporting but a relatively favourable attitude. The determinants of adequate knowledge of ADRs reporting were male gender, working in Ogbomoso zone and training, while the determinant of positive attitude was working in Okeogun zone. Training was the determinant of ADRs reporting among CHEWs. There is an urgent need for educational intervention programmes aiming at increasing the knowledge and modifying the attitude and practices of CHEWs towards increasing ADRs reporting.

### What is known about this topic

- Timely ADRs reporting has contributed immensely towards the prevention of serious ADRs and ensuring public safety;
- Community health extension workers provides basic medical care in rural areas.

### What this study adds

- Majority of the community health extension workers in public health institutions had encountered patients with ADRs but only 26 (12.6%) had reported it with yellow forms;
- Community health extension workers had inadequate knowledge of ADRs reporting;
- The determinant of ADRs reporting among community health wxtension workers was training on ADRs reporting.
